# Preexposure Prophylaxis Among Pregnant and Lactating People in 18 PEPFAR-Supported Countries: A Review of HIV Strategies and Guidelines

**DOI:** 10.9745/GHSP-D-22-00129

**Published:** 2022-12-21

**Authors:** Sheena Sharifi Abadan, Liz Hawryluk, Michele Montandon, Nicole Flowers, Jane Schueller, Robyn Eakle, Pragna Patel, Michelle S. Chevalier, Sangeeta Rana, Anouk Amzel

**Affiliations:** a U.S. Agency for International Development, Washington, DC, USA.; b U.S. Centers for Disease Control and Prevention, Atlanta, GA, USA.; c London School of Hygiene and Tropical Medicine, London, United Kingdom.; d Office of the Global AIDS Coordinator and Health Diplomacy, Washington DC, USA.

## Abstract

**Introduction:**

Pregnant and lactating people (PLP) experience heightened risk of acquiring HIV, which adversely impacts their health and increases the risk for vertical HIV transmission. Preexposure prophylaxis (PrEP), as part of a combination prevention package, including condoms, sexually transmitted infection prevention, and regular HIV testing, is a safe, efficacious method to prevent HIV infections among PLP and their infants. This article examines the evolution of strategies and guidance on PrEP services for PLP from 18 countries supported by the U.S. President’s Emergency Plan for AIDS Relief (PEPFAR).

**Methods:**

The 18 countries implement PEPFAR-supported prevention of vertical transmission of HIV and PrEP programs. We reviewed a total of 18 national HIV strategic plans, 28 national HIV guidelines, and 54 PEPFAR country operational plans (COPs) published in 2013–2020. We compared documents from 2013 to 2017 to those from 2017 to 2020 to assess for differences after the release of the 2017 World Health Organization recommendations supporting the use of PrEP by PLP at substantial risk of acquiring HIV.

**Results:**

National HIV guidelines and PEPFAR COPs that endorsed PrEP for PLP through any categorization increased from 41% to 73% and 11% to 83%, respectively, in the pre-2017 and post-2017 periods. While many documents approved PrEP but not specifically for PLP (10 national strategic plans, 6 national guidelines, and 28 COPs), none of the documents explicitly prohibited PrEP for PLP.

**Conclusion:**

National HIV guidelines and PEPFAR COPs expanded inclusion of PLP in PrEP eligibility when comparing the pre-2017 and the post-2017 groups. However, policy gaps remain as only 36% (4/11) of the post-2017 national HIV guidelines included PLP as a specific priority population for PrEP. Inclusive national HIV strategic plans and guidelines on PrEP for PLP, together with effective program implementation, remain critical for reducing new infections in PLP and eliminating vertical transmission of HIV.

## Abstract (precis)

The use of preexposure prophylaxis (PrEP) is a safe, efficacious method to prevent HIV infections among pregnant and lactating people (PLP) and their infants. Despite growing support for PrEP use among PLP and notable changes in national policies, many policy gaps persist.

## Teaser

**Key Findings:**

- Since the World Health Organization officially endorsed preexposure prophylaxis (PrEP) for pregnant and lactating people (PLP) in 2017, the proportion of national HIV guidelines that endorsed PrEP for PLP under any circumstance increased from 41% before 2017 to 73% in 2017 and after, and the proportion of U.S. President’s Emergency Plan for AIDS Relief country operational plans (COPs) increased from 11% in 2018 to 83% in 2020, with no particular trends by geographic region or prevalence rates.
- Although there has been improvement in inclusion of PLP in PrEP guidelines and strategies, gaps remain. Only 36% of national guidelines published in 2017 or later included PLP specifically as a priority population for PrEP; however, 83% of 2020 PEPFAR COPs included them.
- Having clear global normative guidance based on available evidence provides the opportunity to streamline national policy development, as it can preclude the need for each country to undergo independent research.

**Key Implication:**

- National policy makers, program implementers, and other key stakeholders should continue to develop and operationalize inclusive national HIV strategic plans and guidelines on PrEP for PLP to reduce new infections in PLP and eliminate vertical transmission of HIV.

## INTRODUCTION

Effective HIV prevention services are essential for achieving epidemic control given the estimated 1.7 million new HIV infections still occurring globally as of 2019.^1^ Of those who are newly infected with HIV in sub-Saharan Africa, 63% are women and girls, emphasizing a need for prevention services that are client-controlled, including preexposure prophylaxis (PrEP).^2^

Evidence shows that pregnant and lactating people (previously referred to as pregnant and breastfeeding women) are at up to 2 to 4 times higher risk of acquiring HIV than their nonpregnant and lactating counterparts.^3^ In 21 focus countries in Africa, 27% of new child HIV infections occurred because women newly acquired HIV during pregnancy or breastfeeding.^4^ Globally, about 249 million women of reproductive age (15–49 years) in any given year are pregnant, with 85% receiving facility-based antenatal care (ANC) services.^5^^,^^6^ Furthermore, in the world’s 69 lowest-income countries, 314 million women and girls, a number of whom are lactating, are also accessing modern contraceptive services.^7^

All of these data provide a compelling case that there is a need to provide PrEP to pregnant and lactating people (PLP) to prevent both PLP and their infants from acquiring HIV and that maternal and child health and family planning sites remain important platforms from which PLP could access these services. Further, providing PrEP services at maternal and child health and family planning clinics not only improves access for PLP at risk of acquiring HIV but also for the HIV-negative partners of PLP living with HIV who may visit these sites.

PrEP for PLP was officially endorsed by the World Health Organization (WHO) in 2017, with the publication of a technical brief, Preventing HIV During Pregnancy and Breastfeeding in the Context of PrEP.^8^ In this document, the WHO advocated for access to multiple HIV prevention options for PLP including PrEP, discussed the efficacy of PrEP in preventing maternal and infant HIV infections during pregnancy and lactation, and noted the safety of this drug combination for both women and their infants. Aligned with WHO guidelines, the U.S. President’s Emergency Plan for AIDS Relief (PEPFAR) first discussed PrEP for PLP in its 2018 country operational plan (COP) guidance. Each year, COP guidance documents lay out the most up-to-date parameters and programmatic recommendations for supporting country HIV strategies.^9^ Subsequent COP guidance documents included PLP as a population at risk of acquiring HIV as part of its minimum program requirements for PrEP services in 2020.^10^ Although these documents discuss the most up-to-date programmatic recommendations, they do not assess adoption and implementation of the guidance at the country level.

Aligned with 2017 WHO guidelines, PEPFAR first discussed PrEP for PLP in its 2018 country operational plan guidance.

This article explores the evolution of guidance on PrEP services for PLP from 18 PEPFAR-supported countries through a review of national HIV strategic plans, national HIV guidelines, and PEPFAR COPs. National HIV strategic plans are road maps to guide the national response to the HIV epidemic in a given country over a period of several years. National HIV guidelines include recommendations, protocols, and standard operating procedures for HIV service delivery which are routinely updated. Each PEPFAR country or regional program develops its COP annually, in close collaboration with representatives from host country governments, implementing partners, multilateral bodies, and civil society organizations.

The various sources evaluated for this review were published both before the 2017 WHO guidance was released (2013 to 2017) and after (i.e., from 2017 to 2020), providing an opportunity to understand what, if any, changes in national policies and programs have occurred over time. We hypothesize that the accumulation of evidence on both PrEP safety for PLP and their infants and on the risk of incident infections among PLP—coupled with the provision of relevant global guidance from WHO—has resulted in an enabling environment for policy change and an increase in the number of countries that have incorporated PLP as a priority population for use of PrEP.

## METHODS

Publicly available national HIV strategic plans,^11^^–^^29^ national HIV guidelines,^30^^–^^62^ and PEPFAR COPs^63^^–^^117^ from 18 PEPFAR-supported countries were reviewed to identify countries in which PrEP has been recommended for PLP and under what circumstances. For example, whether PLP had been included as a specific priority population or in limited circumstances, such as only for PLP who are part of serodifferent couples (SDCs), who are adolescent girls and young women (AGYW) at high risk of acquiring HIV, or who are AGYW participating in PEPFAR’s Determined, Resilient, Empowered, AIDS-free, Mentored, and Safe (DREAMS) initiative. In the review, the key search terms used were pregnant and breastfeeding women as the standard language used during the time of these publications.

The 18 countries included in this review were selected because they had PEPFAR-supported programs in both prevention of vertical transmission and PrEP and they had public documents available for analysis. Across these countries, 18 national HIV strategic plans, 28 national HIV and/or PrEP guidelines, and 54 PEPFAR COPs were reviewed. Requirements for inclusion in the document review were that the documents were published between 2013 and 2020 and that they were available in the public domain. The national HIV strategic plans and national HIV guidelines documents were available through ministry of health websites, the Joint United Nations Program on HIV/AIDS website (www.unaids.org), the PrEPWatch website (www.prepwatch.org), and other Internet sites, and the COPs were available through www.pepfar.gov. When reviewing national HIV guidelines, the most recent set of guidelines before 2017 was reviewed and compared to the most recent set of guidelines from 2017 or later. Document collection was conducted by 5 coauthors, 2 of whom conducted the document review.

Across the national HIV strategic plans, national HIV guidelines, and PEPFAR COPs reviewed, we looked for language discussing PrEP for PLP and classified it into the following 6 categories

1. **PrEP specified for PLP under any circumstance (i.e., as a priority population or in limited circumstances):** Any document that included language which recommended PrEP for PLP at all—either as a priority population or in limited circumstances—was included in this category. This category is the summation of documents listed under “PrEP specified for PLP as a priority population” plus “PrEP specified for PLP in limited circumstances” which are further described below:
2. **PrEP specified for PLP as a priority population:** Any document that included language which recommended PrEP for PLP and recognized PLP as a priority population for receiving PrEP was included in this category.
3. **PrEP specified for PLP in limited circumstances:** Any document that included language which recommended PrEP for PLP in limited circumstances, such as through being part of an SDC, being an AGYW at high risk for acquisition of HIV, or being a participant in PEPFAR’s DREAMS initiative.
4. **PrEP specified as a prevention method, but PLP not specified:** Any document that specified PrEP as a prevention tool for HIV but did not explicitly mention PLP was included in this category. This category included documents that mentioned PrEP within pilot projects and/or for key populations (e.g., AGYW, HIV-negative partners in SDCs) but did not explicitly mention PLP.
5. **PrEP specified as a prevention method, but not approved or included for implementation:** Any document that mentioned PrEP but specified that PrEP was not approved or included for implementation was included in this category.
6. **PrEP not specified or mentioned at all:** Any document that did not include mention of PrEP as a prevention method.

Across the documents reviewed, language discussing PrEP for PLP was identified and classified into 6 categories.

In some cases, the relevant document was not available in the public domain or had not been recently updated (e.g., in some countries updated national HIV guidelines have not been released since 2017).

Finally, in discussing the findings from the documents reviewed, we only included those countries where there was implementation of a PrEP program (i.e., countries whose documents were included in the categories of “PrEP specified for PLP under any circumstance as a priority population or in limited circumstances” or “PrEP specified as a prevention method but PLP not specified”). We felt that these countries found PrEP a compelling prevention intervention whether or not they believe PLP to be in the high-risk population categories, while the countries not implementing PrEP were not discerning between populations but about the intervention.

## RESULTS

An in-depth review of relevant national HIV strategic plans and guidelines and PEPFAR COPs was conducted for 18 countries: Botswana, Cameroon, Côte d’Ivoire, Democratic Republic of Congo, Eswatini, Haiti, Kenya, Lesotho, Malawi, Mozambique, Namibia, Nigeria, Rwanda, South Africa, Tanzania, Uganda, Zambia, and Zimbabwe. Overall, 18 national HIV strategic plans, 28 national HIV guidelines (17 before 2017 and 11 from 2017 or later), and 54 COPs were reviewed (Table 1). Although there were many documents in which PrEP for PLP was not specified (10 national strategic plans, 6 national guidelines, and 28 COPs), there were no documents reviewed in this article in which PrEP for PLP was explicitly prohibited.

**TABLE 1.** Review of PrEP for PLP Language in National HIV Strategic Plans, National HIV Guidelines, and PEPFAR COPs^a^

**Country**	**National HIV Strategic Plan** **(Plan Years)**	**National HIV Guidelines 2013–2017** **(Publication Year)**	**National HIV Guidelines 2017–2020** **(Publication Year)**	**COP 2018** **(FY 19)**	**COP 2019** **(FY 20)**	**COP 2020** **(FY 21)**
Botswana	N (2010–2016)^b^	Limited (2016)	N/A	NS	Limited	Y
Cameroon	NS (2018–2022)	X (2015)	N/A	NS	NS	NS
Côte d’Ivoire	N (2015–2019)	N/A	NS (2019)	NS	NS	Y
Democratic Republic of the Congo	N (2018–2021)	Limited (2016)	NS (2018)	N	NS	NS
Eswatini	Y (2018–2023)	N (2015)	N/A	NS	Y	Y
Haiti	NS (2018–2023)	N (2013)	N/A	NS	NS	NS
Kenya	NS (2020–2024)	Limited (2016)	Limited (2018)	NS	NS	Y
Lesotho	Y (2018–2023)	NS (2016)	N/A	NS	Y	Y
Malawi	NS (2015–2020)	X (2016)	Y (2019)	NS	Y	Y
Mozambique	Limited (2020–2024)	N (2015)	N/A	NS	NS	Y
Namibia	Y (2017–2022)	Limited (2016)	Y (2019)	NS	Y	Y
Nigeria	NS (2017–2021)	NS (2016)	N/A	X	NS	Y
Rwanda	NS (2013–2020)	N (2016)	Limited (2018)	NS	NS	Y
South Africa	NS (2017–2022)	Y (2016)	Y (2019)	NS	NS	Y
United Republic of Tanzania	Limited (2017–2022)	N (2015)	NS (2019)	NS	NS	Y
Uganda	NS (2015–2020)	NS (2016)	Limited (2018)	Y	NS	Y
Zambia	NS (2017–2021)	Y (2016)	Y (2020)	Limited	Y	Y
Zimbabwe	NS (2015–2020)	Limited (2016)	Limited (2019)	NS	Y	Y

Abbreviations: COP, country operational plan; DREAMS, Determined, Resilient, Empowered, AIDS-free, Mentored, and Safe; FY, fiscal year; PEPFAR, U.S. President’s Emergency Plan for AIDS Relief; PLP, pregnant and lactating people; PrEP, preexposure prophylaxis; SDC, serodifferent couple.

**^a^**Classification of PrEP for PLP language in these documents: Y, PrEP specified for PLP as a priority population; Limited, PrEP specified for PLP in limited circumstances (SDCs, DREAMS participants, etc.);

NS, PrEP specified as a prevention method, but PLP not specified; X, PrEP specified as a prevention method, but not approved or included for implementation; N, PrEP not specified or mentioned at all; N/A, Document does not exist or was not available.

^b^There was no publicly available national strategic plan that included the time period after 2017.

Here we review the findings and changes over time in each of the 3 document types: the national strategic plans; the pre- and post-2017 national guidelines; and the 3 years of COPs—2018, 2019, and 2020.

### National HIV Strategic Plans

All 18 countries had available national HIV strategic plans that were reviewed. Fifteen of 18 countries recommended PrEP as a prevention tool, and 3 countries (Botswana, Cote d’Ivoire, and the Democratic Republic of Congo) did not specify or mention PrEP at all. Three countries (Eswatini, Lesotho, and Namibia) recognized PLP as a priority population for PrEP, and 2 others (Mozambique and United Republic of Tanzania) specified PrEP for PLP in limited circumstances. While 10 countries (Cameroon, Haiti, Kenya, Malawi, Nigeria, Rwanda, South Africa, Uganda, Zambia, and Zimbabwe) did not mention PrEP for PLP specifically in their national HIV strategic plans, none of the documents reviewed explicitly prohibited this population from accessing PrEP.

While 10 countries did not mention PrEP for PLP specifically in their national HIV strategic plans, none of the documents reviewed explicitly prohibited PLP from accessing PrEP.

Table 2 provides a review of the above findings related to national HIV strategic plans.

**TABLE 2.** Review of National HIV Strategic Plans

**Document Categories**	**Countries, %(No.)**
PrEP specified for PLP under any circumstance (i.e., as a priority population or in limitedcircumstances)	28 (5)
PrEP specified for PLP as a priority population	17 (3)^a^
PrEP specified for PLP in limited circumstances	11 (2)^b^
PrEP specified as a prevention method but PLP not specified	56 (10)^c^
PrEP specified as a prevention method but not approved or included for implementation	0
PrEP not specified or mentioned at all	17 (3)^d^
Total reviewed	18

Abbreviation: PLP, pregnant and lactating people; PrEP, preexposure prophylaxis.

a Eswatini, Lesotho, and Namibia.

b Mozambique and United Republic of Tanzania.

c Cameroon, Haiti, Kenya, Malawi, Nigeria, Rwanda, South Africa, Uganda, Zambia, and Zimbabwe.

d Botswana, Cote d’Ivoire, and Democratic Republic of the Congo.

### National HIV Guidelines

Overall, there were 17 available national HIV guidelines published before 2017 and 11 from 2017 or after. Notably, Côte d’Ivoire did not have guidelines before 2017 publicly available within the 6-year time frame, and 7 countries (Botswana, Cameroon, Eswatini, Haiti, Lesotho, Mozambique, Nigeria) did not have guidelines publicly available from 2017 or later. Of the 17 pre-2017 guidelines reviewed, 12 included PrEP as a prevention method, while all 11 of the post-2017 guidelines assessed espoused PrEP.

Of those 12 countries whose pre-2017 guidelines included PrEP, only 2 (South Africa, Zambia) included mention of PLP specifically as a priority population for PrEP—both with caveats. In Zambia’s guidelines, PrEP can be provided to this population specifically if the prescribing clinician believes the risk of HIV seroconversion in a PLP is high. While in the South African pre-2017 guidelines, there is a note that PrEP in PLP is contraindicated due to limited safety data; however, the guidelines do also note that given the high risk of seroconversion in this population, risks and benefits should be discussed and PLP should be allowed to make an informed decision for themselves. An additional 5 countries (Botswana, Democratic Republic of the Congo, Kenya, Namibia, and Zimbabwe) specified that PLP could access PrEP only in limited circumstances, most frequently as part of an SDC. Therefore, a total of 7 of the 12 countries noted that PrEP could be used in PLP under any circumstances before 2017.

All 11 post-2017 national guidelines included PrEP as an HIV prevention method. Furthermore, PLP were explicitly listed as a priority population for PrEP in 4 (Malawi, Namibia, South Africa, and Zambia) of the 11 guidelines, a notable doubling from the pre-2017 group. National HIV guidelines specified PrEP eligibility for PLP in limited circumstances (e.g., as part of an SDC) in 4 (Kenya, Rwanda, Uganda, and Zimbabwe) of the 11 post-2017 guidelines, for a total of 8 of the 11 post-2017 national guidelines endorsing PrEP for PLP.

Table 3 provides a review of the above findings related to national HIV guidelines.

**TABLE 3.** Review of National HIV Guidelines, Pre-2017 and Post-2017

**Document Categories**	**2013–2017, % (No.)**	**2017–2020, % (No.) **
PrEP specified for PLP under any circumstance (i.e., as a priority population or in limited circumstances)	41 (7)	73 (8)
PrEP specified for PLP as a priority population	12 (2)^a^	36 (4)^b^
PrEP specified for PLP in limited circumstances	29 (5)^c^	36 (4)^d^
PrEP specified as a prevention method but PLP not specified	18 (3)^e^	27 (3)^f^
PrEP specified as a prevention method but not approved or included for implementation	12 (2)^g^	0
PrEP not specified or mentioned at all	29 (5)^h^	0
Total reviewed	17	11

Abbreviations: PLP, pregnant and lactating people; PrEP, preexposure prophylaxis.

^a^South Africa and Zambia.

^b^Malawi, Namibia, South Africa, and Zambia.

^c^Botswana, Democratic Republic of the Congo, Kenya, Namibia, and Zimbabwe.

^d^Kenya, Rwanda, Uganda, and Zimbabwe.

^e^Lesotho, Nigeria, and Uganda.

^f^Cote d’Ivoire, Democratic Republic of the Congo, and United Republic of Tanzania.

^g^Cameroon and Malawi.

^h^Eswatini, Haiti, Mozambique, Rwanda, and United Republic of Tanzania.

### PEPFAR COPs

The PEPFAR COPs from 2018, 2019, and 2020 were reviewed for the 18 selected countries (Table 4). These documents represent the final decisions agreed upon for the yearly strategic direction of PEPFAR support to the national country's HIV programs. They are typically published between March and May of the same calendar year. In COPs from 2018, only Uganda recommended PrEP for PLP as a priority population. Additionally, for 2018, while Nigeria mentioned PrEP in its COP, it did not include PrEP as part of prevention strategies to be implemented. In COPs from 2019, 6 of the 18 countries (Eswatini, Lesotho, Malawi, Namibia, Zambia, and Zimbabwe) recommended PrEP for PLP as a priority population at risk of acquiring HIV. In COPs from 2020, 15 out of 18 countries recommended PrEP for PLP as a priority population. Cameroon, Côte d’Ivoire, and the Democratic Republic of the Congo did not specify PLP as a population eligible for PrEP, although HIV-negative partners within SDCs were mentioned as eligible for PrEP. Of interest, Zimbabwe further designated distinct PrEP targets for PLP in their COP for 2020.

**TABLE 4.** Review of PEPFAR COPs, 2018–2020

**Document Categories**	**2018, % (No.)**	**2019, % (No.)**	**2020, % (No.)**
PrEP specified for PLP under any circumstance (i.e., as a priority population or in limited circumstances)	11 (2)	39 (7)	83 (15)
PrEP specified for PLP as a priority population	6 (1)^a^	33 (6)^b^	83 (15)^c^
PrEP specified for PLP in limited circumstances	6 (1)^d^	6 (1)^e^	0
PrEP specified as a prevention method but PLP not specified	78 (14)^f^	61 (11)^g^	17 (3)^h^
PrEP specified as a prevention method but not approved or included for implementation	6 (1)^i^	0	0
PrEP not specified or mentioned at all	6 (1)^j^	0	0
Total reviewed, No.	18	18	18

Abbreviations: COP, country operational plan; PLP, pregnant and lactating people; PEPFAR, U.S. President’s Emergency Plan for AIDS Relief; PrEP, preexposure prophylaxis.

a Uganda.

b Eswatini, Lesotho, Malawi, Namibia, Zambia, and Zimbabwe.

c Botswana, Cote d’Ivoire, Eswatini, Kenya, Lesotho, Malawi, Mozambique, Namibia, Nigeria, Rwanda, South Africa, United Republic of Tanzania, Uganda, Zambia, and Zimbabwe.

d Zimbabwe.

e Botswana.

f Botswana, Cameroon, Cote d’Ivoire, Eswatini, Haiti, Kenya, Lesotho, Malawi, Mozambique, Namibia, Rwanda, South Africa, United Republic of Tanzania, and Zimbabwe.

g Cameroon, Cote d’Ivoire, Democratic Republic of the Congo, Haiti, Kenya, Mozambique, Nigeria, Rwanda, South Africa, United Republic of Tanzania, and Uganda.

h Cameroon, Democratic Republic of the Congo, and Haiti.

i Nigeria.

j Democratic Republic of the Congo.

## DISCUSSION

In this review of national HIV strategic plans, national HIV guidelines, and PEPFAR COPs, we observed an increase in the proportion of national guidelines and COPs that addressed PrEP for PLP over time. The proportion of national HIV guidelines that endorsed PrEP for PLP under any circumstance increased from 41% pre-2017 to 73% post-2017, while the proportion of PEPFAR COPs increased from 11% in 2018 to 83% in 2020. Only 1 national HIV strategic plan was reviewed per country, and these plans cover longer periods of time, so changes over time could not be assessed. Of the 18 national HIV strategic plans analyzed, only 28% discussed PrEP for PLP. It was interesting to note that there do not seem to be particular trends by geographic or prevalence rates for adoption and inclusion of PLP as a high-risk population eligible for PrEP. Looking at the national strategic plans, it would appear that southern African countries were early adopters; however, these countries’ plans were also written at a later date than most. There was a varied mix of countries pre-2017 that incorporated the WHO guidance into their guidelines—but all with caveats, some stronger (South Africa and Zambia) than others (Botswana, Democratic Republic of the Congo, Kenya, Namibia, and Zimbabwe). No true patterns in uptake emerged.

We observed an increase in the proportion of national guidelines and COPs that addressed PrEP for PLP over time.

It is important to note that long-acting cabotegravir and the dapivirine ring were not options for PrEP when these countries were developing the strategic plans and guidance documents reviewed in this article, so its findings reflect the perspective of use of oral PrEP only.

Over time, as critical evidence and global guidance supporting PrEP use among PLP grew, countries revised their national HIV guidelines and PEPFAR teams revised their COPs to include PrEP as a prevention strategy for PLP. Having clear global normative guidance based on available evidence provides the opportunity to streamline national policy development, as it can preclude the need for each country to undergo independent research, evaluate the significance of their findings, and develop policies based on these findings. Additionally, both newly available evidence and normative guidance can catalyze in-country stakeholders, especially civil society organizations, to begin the dialogue advocating for changes in national policies. The results of this review point to policies and/or guidelines being updated and adopted by additional countries after the 2017 WHO recommendations endorsing PLP as a PrEP priority population were published.

Although there has been improvement in inclusion of PLP in PrEP guidelines and strategies, gaps remain. Only 36% (4/11) of national guidelines published in 2017 or later included PLP specifically as a priority population for PrEP (i.e., not just as a part of SDC, an AGYW, or the DREAMS program). However, 15 of 18 (83%) 2020 PEPFAR COPs included them. This finding is not a surprise as countries have different ways of disseminating changes in guidance that are not always full guidance reviews, and differing document emphases can reflect in language variances.

Recent evidence from the PrEP Implementation for Mothers in Antenatal Care study in Kenya found a substantial proportion of pregnant women were at risk for HIV, and that targeted, risk-based PrEP offer did not improve PrEP decision making or decrease HIV incidence compared to universal PrEP counseling and offer of PrEP.^118^ Therefore, limiting PrEP use in PLP to subpopulations, such as partners in known SDCs or at-risk AGYW, may reduce PrEP access for women who are at risk of HIV acquisition and also complicates the eligibility assessment for PrEP service delivery. Findings of increased incidence of HIV in PLP overall as well as implementation experience in providing PrEP to PLP supports expanded guidelines and strategies for offering PrEP counseling and services to all PLP in high HIV burden settings, rather than solely focusing on subpopulations.^119^

### Limitations

There were limitations to our review. Only publicly available national guidelines, national strategic plans, and PEPFAR COPs were included in this analysis; however, some countries have more current policy documents and PrEP implementation guides that were not included in this review. We also acknowledge that revising and updating national HIV strategic plans and guidelines can be a lengthy and complicated process involving numerous stakeholders. This often results in ministries of health using other forms of documentation, such as a circular or memo, to quickly communicate changes in policies and guidelines. These other forms of guidance change communication were not included in this review.

Another limitation of this review is that our policy analysis did not allow us to assess how these strategy documents have been implemented, nor did our methodology allow us to gain insight into challenges, partial successes, outcomes, or impact. An enabling policy environment is necessary but not sufficient for effective, wide-scale implementation of PrEP for PLP. There are known delays in translating research into practice, both in incorporating research findings into guidelines and then in moving from guidelines to operationalization. Barriers to implementing PrEP for PLP can include limitations in service provider capacity, limited resources for scale-up, restrictive social and gender norms, and potential client and provider concerns about side effects, including those related to fetal/infant safety.^120^^,^^121^ Additional research is needed to explore how existing policies, strategic plans, and guidelines are being implemented with fidelity; national stakeholders’ role in transitioning national guidance when new evidence and normative guidelines emerge; and the potential impact of the newer PrEP formulations of cabotegravir and the dapivirine ring.

## CONCLUSION

There was an increase in the proportion of national HIV guidelines and PEPFAR COPs that recommended PrEP for PLP in the years after the release of supportive global WHO guidelines but gaps in the published national guidelines persist in many countries. Inclusive national HIV strategic plans and guidelines on PrEP for PLP, as well as effective program implementation and incorporation of newer PrEP formulations, are critical for reducing new infections in PLP and eliminating vertical transmission of HIV.

## Notes

**Peer Reviewed**

**First published online:** December 1, 2022.
